# A cAbl-MRTF-A Feedback Loop Contributes to Hepatic Stellate Cell Activation

**DOI:** 10.3389/fcell.2019.00243

**Published:** 2019-10-16

**Authors:** Yunjie Lu, Fangqiao Lv, Ming Kong, Xuyang Chen, Yunfei Duan, Xuemin Chen, Donglin Sun, Mingming Fang, Yong Xu

**Affiliations:** ^1^Department of Hepatobiliary and Pancreatic Surgery, The First People’s Hospital of Changzhou, The Third Affiliated Hospital of Soochow University, Changzhou, China; ^2^Department of Cell Biology, School of Basic Medical Sciences, Capital Medical University, Beijing, China; ^3^Key Laboratory of Targeted Intervention of Cardiovascular Disease, Collaborative Innovation Center for Cardiovascular Disease Translational Medicine, Department of Pathophysiology, Nanjing Medical University, Nanjing, China; ^4^Institute of Biomedical Research, Liaocheng University, Liaocheng, China; ^5^Department of Clinical Medicine and Laboratory Center for Experimental Medicine, Jiangsu Vocational College of Medicine, Nanjing, China

**Keywords:** transcriptional regulation, hepatic stellate cell, post-translational modification, signal transduction, liver fibrosis

## Abstract

Trans-differentiation of quiescent hepatic stellate cells (HSC) to myofibroblasts is a hallmark event in liver fibrosis. Previous studies have led to the discovery that myocardin-related transcription factor A (MRTF-A) is a key regulator of HSC trans-differentiation or, activation. In the present study we investigated the interplay between MRTF-A and c-Abl (encoded by *Abl1*), a tyrosine kinase, in this process. We report that hepatic expression levels of c-Abl were down-regulated in MRTF-A knockout (KO) mice compared to wild type (WT) littermates in several different models of liver fibrosis. MRTF-A deficiency also resulted in c-Abl down-regulation in freshly isolated HSCs from the fibrotic livers of mice. MRTF-A knockdown or inhibition repressed c-Abl in cultured HSCs *in vitro*. Further analyses revealed that MRTF-A directly bound to the *Abl1* promoter to activate transcription by interacting with Sp1. Reciprocally, pharmaceutical inhibition of c-Abl suppressed MRTF-A activity. Mechanistically, c-Abl activated extracellular signal-regulated kinase (ERK), which in turn phosphorylated MRTF-A and promoted MRTF-A nuclear trans-localization. In conclusion, our data suggest that a c-Abl-MRTF-A positive feedback loop contributes to HSC activation and liver fibrosis.

## Introduction

Liver fibrosis is generally perceived as a host defense mechanism in response to a myriad of danger signals that include pathogens, toxins, and nutrients/metabolites (Duffield et al., 2013). The failure to terminate fibrogenesis after the clearance of the triggering danger signals invariably causes structural and functional disturbances to the liver and is associated with end-stage liver diseases (Friedman, 2010). Liver fibrosis parallels proliferation and migration of myofibroblasts, which produce and secret extracellular matrix (ECM) proteins such as collagen type I, collagen type III, and fibronectin (Kisseleva, 2017). Myofibroblasts are typically characterized by the expression of signature genes including alpha smooth muscle actin (α-SMA). Origins of hepatic myofibroblasts have been a topic of heated debate although recent fate-mapping experiments suggest that hepatic stellate cells (HSCs) are the predominant source for activated myofibroblasts in the fibrotic liver independent of the etiology (Mederacke et al., 2013). HSCs adopt a quiescent state in the normal liver functioning primarily as a reservoir for certain lipids and vitamins. Once challenged with physical (e.g., alterations of matrix stiffness) and biochemical (e.g., transforming growth factor/TGF and platelet derived growth factor/PDGF) cues, HSCs trans-differentiate into myofibroblasts to promote liver fibrosis (Tsukamoto et al., 2011).

Myocardin-related transcription factor A (MRTF-A) is the master regulator of myofibroblast maturation. Mounting evidence supports a pivotal role for MRTF-A in bridging cytoskeletal remodeling to cellular response to mechanostress (Olson and Nordheim, 2010). An array of sequence-specific transcription factors involved in liver fibrosis, including SMAD (Morita et al., 2007), SRF (Small et al., 2010), YAP/TAZ (Piersma et al., 2015), and Sp1 (Luchsinger et al., 2011), have been reported to interact with MRTF-A to program the fibrogenic response. The activity of MRTF-A is subjected to several layers of regulation. A number of stress stimuli, including hypoxia, inflammation, and reactive oxygen species (ROS), can up-regulate MRTF-A expression (Yang et al., 2014; Chen et al., 2015; Yu et al., 2017a, 2018). MRTF-A can also be shuttled between the cytoplasm and the nucleus, where it exerts the regulatory functions, in a RhoA-dependent manner (Posern and Treisman, 2006). Post-translational modifications, such as phosphorylation (Panayiotou et al., 2016) and acetylation (Yu et al., 2017b), have been shown to modulate MRTF-A activity by affecting its sub-cellular localization and/or protein-protein interactions.

c-Abl (encoded by *Abl1*) is a member of the Abelson family of non-receptor kinases playing key roles in diverse cellular processes (Khatri et al., 2016). c-Abl activation is frequently found in the process of cellular fibrogenesis. For instance, Bujor et al. (2011) have reported that activation of c-Abl by TGF-β regulates phosphorylation of Fli-1, a pro-fibrogenic transcription factor, in dermal fibroblasts to promote the synthesis of collagens. Ceni et al. (2006) have shown that c-Abl is activated by H_2_O_2_ and is essential for ROS-induced activation of human HSCs. On the contrary, c-Abl inhibitors attenuate organ fibrosis in several different animal models (Wang et al., 2005; Akhmetshina et al., 2008; Liu et al., 2011). Of interest, c-Abl expression levels are elevated in fibrotic tissues although the mechanism is not clear (Karimizadeh et al., 2015). In the present study we investigated the interplay between MRTF-A and c-Abl during HSC activation. We report that MRTF-A directly binds to the c-Abl promoter to activate c-Abl transcription in activated HSCs. Reciprocally c-Abl activates ERK, which in turn phosphorylates MRTF-A and promotes MRTF-A nuclear trans-location. We therefore propose that a c-Abl-MRTF-A positive feedback loop contributes to HSC activation.

## Materials and Methods

### Cell Culture, Plasmids, Transient Transfection, and Reporter Assay

Immortalized human hepatic stellate cells (LX-2) and primary hepatic stellate cells were maintained previously described. FLAG-tagged MRTF-A expression constructs, Myc-tagged c-Abl expression constructs, HA-tagged Sp1 expression constructs, the *ACTA2* promoter-luciferase construct, and the *COL1A2* promoter-luciferase construct have been previously described (Cong et al., 2000; Sun et al., 2013; Fan et al., 2015; Li et al., 2018a). The human c-Abl promoter-luciferase construct was made by amplifying ∼1.4 kb of genomic DNA spanning the proximal ABL1 promoter (−1204/+183) and ligating the amplicon into the pGL4 vector (Promega). Mutation constructs were made using the QuikChange Mutagenesis Kit (Agilent). Cells were harvested 48 h after transfection and reporter activity was measured using a luciferase reporter assay system (Promega) as previously described (Li et al., 2018b).

### Protein Extraction and Western Blot

Whole cell lysates were obtained by re-suspending cell pellets in RIPA buffer (50 mM Tris pH7.4, 150 mM NaCl, 1% Triton X-100) with freshly added protease inhibitor (Roche) as previously described (Zeng et al., 2018). Western blot analyses were performed with anti-MRTF-A (Santa Cruz, sc-32909), anti-phosphoserine (Abcam, ab9332), anti-phosphotyrosine (Abcam, ab10321), anti-c-Abl (Santa Cruz, sc-131), anti-α-SMA (Sigma, A2547), anti-Sp1 (Santa Cruz, sc-14027), and anti-β-actin (Sigma, A2228) antibodies. All experiments were repeated three times.

### RNA Isolation and Real-Time PCR

RNA was extracted with the RNeasy RNA isolation kit (Qiagen) as previously described (Shao et al., 2019). Reverse transcriptase reactions were performed using a SuperScript First-Strand Synthesis System (Invitrogen). Real-time PCR reactions were performed on an ABI Prism 7500 system. Primers and TaqMan probes used for real-time reactions were purchased from Applied Biosystems. All experiments were performed in triplicate wells (technical replicates) and repeated at least three times. Data are presented as mean ± SD.

### Chromatin Immunoprecipitation

Chromatin Immunoprecipitation (ChIP) assays were performed essentially as described before (Weng et al., 2019; Yang et al., 2019). In brief, chromatin in control and treated cells were cross-linked with 1% formaldehyde. Cells were incubated in lysis buffer (150 mM NaCl, 25 mM Tris pH 7.5, 1% Triton X-100, 0.1% SDS, 0.5% deoxycholate) supplemented with protease inhibitor tablet and PMSF. DNA was fragmented into <500 bp pieces using a Branson 250 sonicator. Aliquots of lysates containing 200 μg of protein were used for each immunoprecipitation reaction with anti-MRTF-A (Santa Cruz, sc-32909), pre-immune IgG. For re-ChIP, immune complexes were eluted with the elution buffer (1% SDS, 100 mM NaCO_3_), diluted with the re-ChIP buffer (1% Triton X-100, 2 mM EDTA, 150 mM NaCl, 20 mM Tris pH 8.1), and subject to immunoprecipitation with a second antibody of interest. Precipitated genomic DNA was amplified by real-time PCR with the following primers: All experiments were performed in triplicate wells (technical replicates) and repeated at least three times. Data are presented as mean ± SD.

### Animals

All animal experiments were reviewed and approved by the intramural Ethics Committee on Humane Treatment of Experimental Animals. The breedings were conducted by Nanjing Biomedical Research Institute of Nanjing University. The global MRTF-A knockout mice were kindly provided by Steve Morris at St Jude’s Hospital (Sun et al., 2006). To induce liver fibrosis, 6–8 week-old male C57/BL6 mice (WT and sex/age-matched MRTF-A KO) were injected with CCl_4_ (1.0 mL/kg body weight as 50%, vol/vol) once a week for 6 weeks. Alternatively, mice were injected with concanavalin A (100 mg/kg body weight) every other day for 2 weeks. In a third model, the common bile duct was ligated twice with silk sutures. Bile duct ligation (BDL) and sham-operated mice were sacrificed 2 weeks following the surgical procedure.

### Immunofluorescence Microscopy

The cells were fixed with 4% formaldehyde, permeabilized with TBST (0.25% Triton X-100, 150 mM NaCl, 50 mM Tris pH7.4), blocked with 5% BSA, and incubated with indicated primary antibodies overnight. After several washes with PBS, cells were incubated with FITC-labeled secondary antibodies (Jackson) for 30 min. DAPI (Sigma) was added and incubated with cells for 5 min prior to observation. Immunofluorescence was visualized on a co-focal microscope (LSM 710, Zeiss). For each group, at least 10 fields were counted.

### Statistical Analysis

One-way ANOVA with *post hoc* Scheffe analyses were performed using an SPSS package. Data are presented as mean ± SD. *P* values smaller than 0.05 were considered statistically significant (^∗^).

## Results

### MRTF-A Regulates Abl1 Expression in the Fibrotic Liver *in vivo*

**FIGURE 1:**
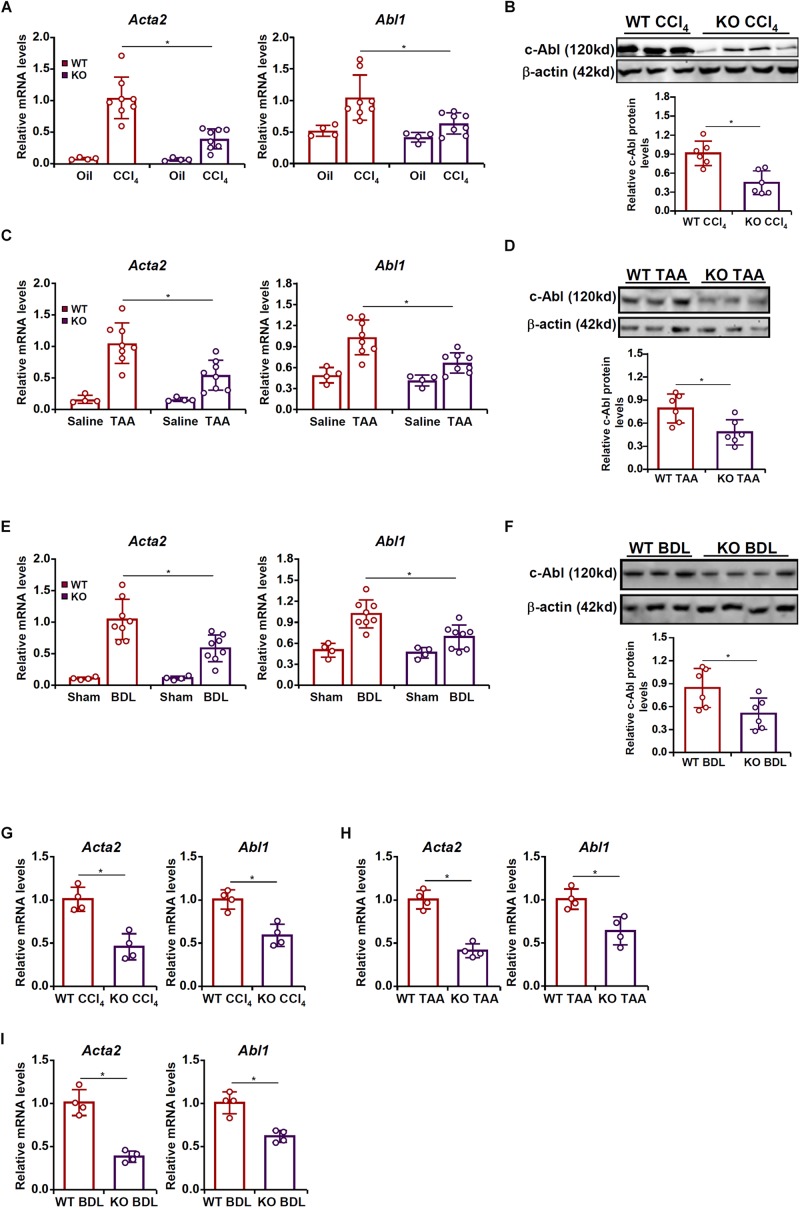
MRTF-A regulates Abl1 expression in the fibrotic liver *in vivo*. **(A,B)** Wild type (WT) and MRTF-A knockout (KO) mice were injected with CCl_4_ to induce liver fibrosis as described in section “Materials and Methods.” Gene expression levels were examined by qPCR and Western. *N* = 4 mice for the corn oil groups and *N* = 8 mice for the CCl_4_ groups. **(C,D)** WT and MRTF-A KO mice were injected with TAA to induce liver fibrosis as described in section “Materials and Methods.” Gene expression levels were examined by qPCR and Western. *N* = 4 mice for the saline groups and *N* = 8 mice for the TAA groups. **(E,F)** WT and MRTF-A KO mice were subjected to bile duct ligation (BDL) to induce liver fibrosis as described in section “Materials and Methods.” Gene expression levels were examined by qPCR and Western. *N* = 4 mice for the sham groups and *N* = 8 mice for the BDL groups. **(G)** WT and MRTF-A KO mice were injected with CCl_4_ to induce liver fibrosis as described in section “Materials and Methods.” Primary HSCs were isolated and gene expression levels were examined by qPCR. *N* = 4 mice for each group. **(H)** WT and MRTF-A KO mice were injected with TAA to induce liver fibrosis as described in section “Materials and Methods.” Primary HSCs were isolated and gene expression levels were examined by qPCR. *N* = 4 mice for each group. **(I)** WT and MRTF-A KO mice were subjected to BDL to induce liver fibrosis as described in section “Materials and Methods.” Primary HSCs were isolated and gene expression levels were examined by qPCR. *N* = 4 mice for each group. All experiments were repeated three times and one representative experiment is shown. Error bars represent SD (^∗^*p* < 0.05, one-way ANOVA with *post hoc* Scheffe test).

To examine the potential MRTF-A-c-Abl interplay in HSC activation and liver fibrosis, the following experiments were performed. Liver fibrosis was induced in wild type (WT) and MRTF-A knockout (KO) mice by several different methods. In the first model, the mice were injected with CCl_4_ for 6 weeks. Consistent with previously published data, MRTF-A deletion attenuated liver fibrosis as indicated by the down-regulation of α-SMA (*Acta2*) expression. Of interest, MRTF-A deficiency decreased hepatic c-Abl (*Abl1*) levels (Figures 1A,B). In the second model, the mice were injected with TAA for 2 weeks. MRTF-A loss-of-function led to decreased expression of both α-SMA and c-Abl in the liver (Figures 1C,D). In the third model, the mice were subjected to the BDL procedure. Again, a simultaneous amelioration of α-SMA and c-Abl was detected in the KO livers compared to the WT livers (Figures 1E,F).

Next, HSCs were freshly isolated from the fibrotic livers of the WT mice and the KO mice. Quantitative PCR confirmed that MRTF-A deficiency diminished c-Abl expression in HSCs in the CCl4 model (Figure 1G), the TAA model (Figure 1H), and the BDL model (Figure 1I).

### MRTF-A Regulates Abl1 Expression in Activated Hepatic Stellate Cells *in vitro*

**FIGURE 2:**
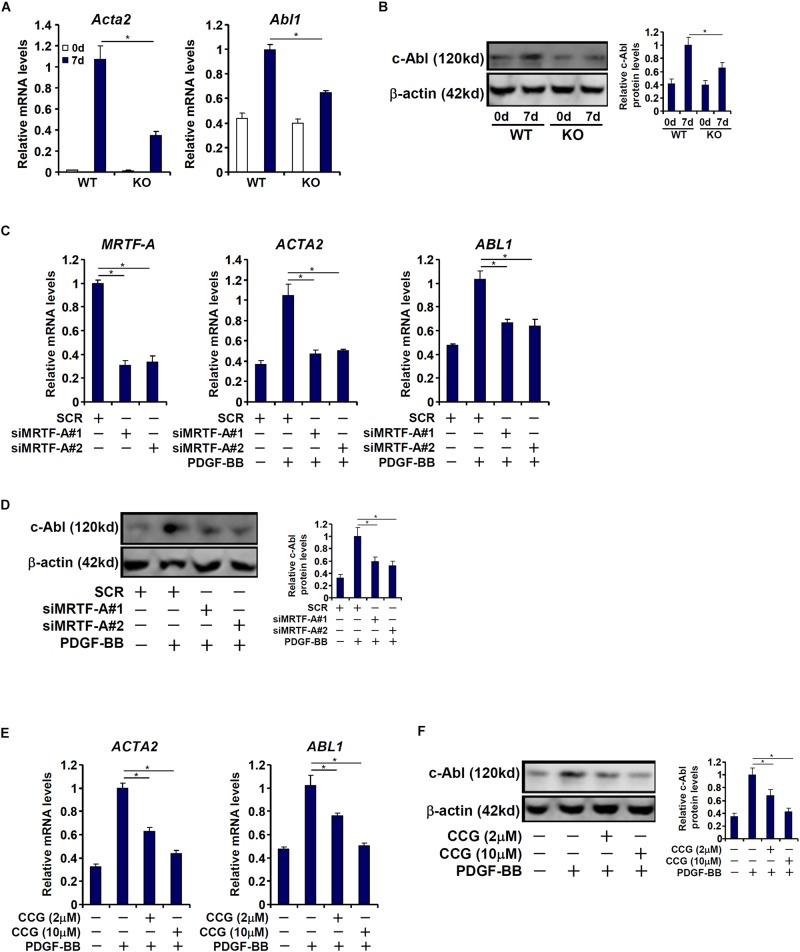
MRTF-A regulates Abl1 expression in activated hepatic stellate cells *in vitro*. **(A,B)** Primary HSCs isolated from WT and MRTF-A KO mice were spontaneously activated in culture for 7 days. Gene expression levels were examined by qPCR and Western. **(C,D)** LX-2 cells were transfected with small interfering RNA targeting MRTF-A or scrambled siRNA (SCR) followed by treatment with PDGF-BB. Gene expression levels were examined by qPCR and Western. **(E,F)** LX-2 cells were treated with PDGF-BB in the presence or absence of CCG-1423 (1–10 μM). Gene expression levels were examined by qPCR and Western. All experiments were repeated three times and one representative experiment is shown. Error bars represent SD (^∗^*p* < 0.05, one-way ANOVA with *post hoc* Scheffe test).

We then attempted to verify whether MRTF-A might regulate c-Abl expression in cultured HSCs *in vitro*. To this end, primary HSCs were isolated from the WT and the KO mice and allowed to undergo spontaneous activation. As shown in Figures 2A,B, c-Abl expression was augmented in activated HSCs compared to quiescent HSCs; the induction of c-Abl expression was more robust in WT cells than in KO cells. Next, MRTF-A was depleted in human HSCs (LX-2) by small interfering RNAs (siRNAs). MRTF-A knockdown significantly suppressed the induction of c-Abl expression by PDGF treatment (Figures 2C,D). Similarly, treatment of CCG-1423, a small-molecule MRTF-A inhibitor, alleviated c-Abl induction by PDGF in a dose-dependent manner (Figures 2E,F). Combined, these data suggest that MRTF-A regulates c-Abl expression during HSC activation both *in vivo* and *in vitro*.

### MRTF-A Activates Abl1 Transcription in Hepatic Stellate Cells

**FIGURE 3:**
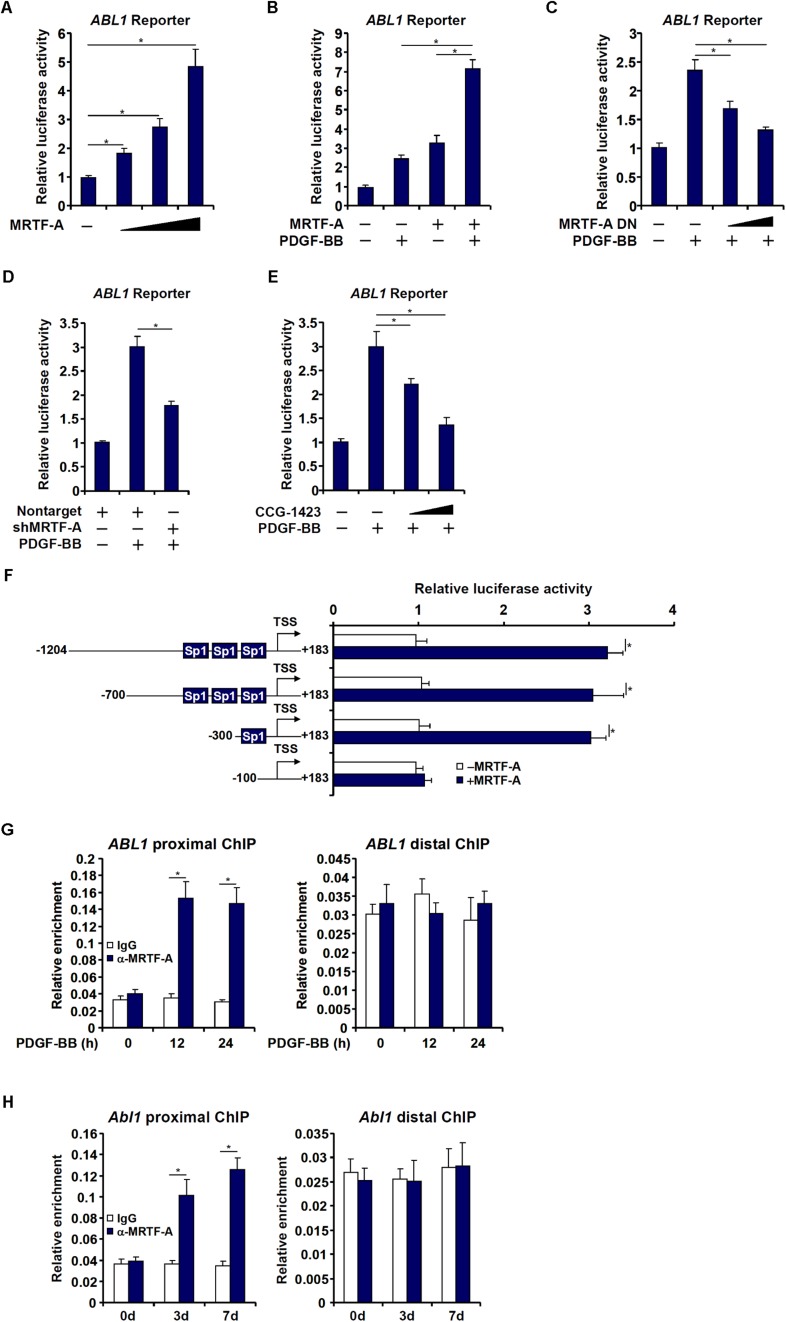
MRTF-A activates Abl1 transcription in hepatic stellate cells. **(A)** An Abl1 promoter-luciferase construct was transfected into LX-2 cells with increasing doses of MRTF-A. Luciferase activities were normalized by both protein concentration and GFP fluorescence. **(B)** An Abl1 promoter-luciferase construct was transfected into LX-2 cells with or without MRTF-A followed by treatment with PDGF. Luciferase activities were normalized by both protein concentration and GFP fluorescence. **(C)** An Abl1 promoter-luciferase construct was transfected into LX-2 cells with a dominant negative (DN) MRTF-A followed by treatment with PDGF. Luciferase activities were normalized by both protein concentration and GFP fluorescence. **(D)** An Abl1 promoter-luciferase construct was transfected into LX-2 cells with a short hairpin RNA (shRNA) plasmid targeting MRTF-A followed by treatment with PDGF. Luciferase activities were normalized by both protein concentration and GFP fluorescence. **(E)** An Abl1 promoter-luciferase construct was transfected into LX-2 cells followed by treatment with PDGF and/or CCG-1423 (1–10 μM). Luciferase activities were normalized by both protein concentration and GFP fluorescence. **(F)** Abl1 promoter-luciferase constructs of various lengths were transfected into LX-2 cells with or without MRTF-A. Luciferase activities were normalized by both protein concentration and GFP f luorescence. **(G)** Primary HSCs were spontaneously activated in culture for 7 days. ChIP assays were performed with anti-MRTF-A or IgG. **(H)** LX-2 cells were treated with PDGF and harvested at indicated time points. ChIP assays were performed with anti-MRTF-A or IgG. All experiments were repeated three times and one representative experiment is shown. Error bars represent SD (^∗^*p* < 0.05, one-way ANOVA with *post hoc* Scheffe test).

Next, we investigated whether MRTF-A could directly bind to the c-Abl promoter and activate c-Abl transcription. To this end, a human *ABL1* promoter reporter (−1204/+183) was transfected into LX-2 cells. Over-expression of MRTF-A dose dependently activated the c-Abl promoter (Figure 3A), indicating that MRTF-A regulates c-Abl expression primarily at the transcriptional level. MRTF-A over-expression also enhanced the activation of the c-Abl promoter by PDGF treatment (Figure 3B). Consistent with this observation, over-expression of a dominant negative (DN) MRTF-A abolished the activation of the c-Abl promoter by PDGF (Figure 3C). Short hairpin RNA (shRNA) plasmid targeting MRTF-A achieved similar effects as the DN MRTF-A (Figure 3D). Pharmaceutical inhibition of MRTF-A with CCG-1423 comparably suppressed the activation of the c-Abl promoter by PDGF (Figure 3E). In order to pin down the region to which MRTF-A binds on the c-Abl promoter, a series of inward deletions were introduced to the full-length *ABL1* promoter construct (Figure 3F). MRTF-A was able to activate the c-Abl promoter until the deletion went beyond −100 relative to the transcription start site (TSS), suggesting that MRTF-A might occupy somewhere between −100 and −300 of the proximal c-Abl promoter. Indeed, ChIP assays performed in both LX-2 cells (Figure 3G) and primary mouse HSCs (Figure 3H) confirmed that association of MRTF-A with the proximal c-Abl promoter, but not the distal c-Abl promoter, was significantly augmented during HSC activation.

**FIGURE 4:**
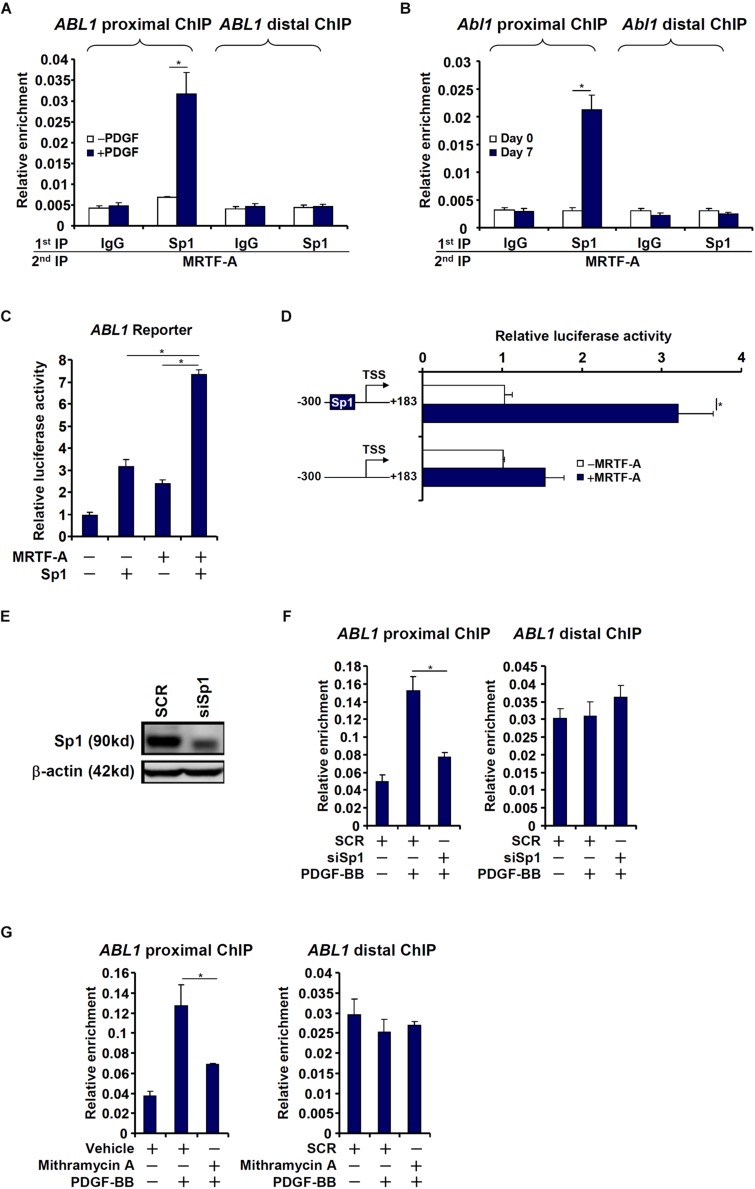
MRTF-A interacts with Sp1 to activate Abl1 transcription in hepatic stellate cells. **(A)** LX-2 cells were treated with or without PDGF-BB for 24 h. Re-ChIP assay was performed with indicated antibodies. **(B)** Primary mouse HSCs were isolated and spontaneously activated in culture for 7 days. Re-ChIP assay was performed with indicated antibodies. **(C)** An Abl1 promoter-luciferase construct was transfected into LX-2 cells with indicated expression constructs. Luciferase activities were normalized by both protein concentration and GFP fluorescence. **(D)** Wild type or mutant Abl1 promoter-luciferase constructs were transfected into LX-2 cells with or without MRTF-A. Luciferase activities were normalized by both protein concentration and GFP fluorescence. **(E,F)** LX-2 cells were transfected with small interfering RNA targeting Sp1 or SCR followed by treatment with PDGF-BB. Knockdown efficiencies were examined by Western. ChIP assays were performed with anti-MRTF-A. **(G)** LX-2 cells were with PDGF-BB and/or mithramycin A for 24 h. ChIP assays were performed with anti-MRTF-A. All experiments were repeated three times and one representative experiment is shown. Error bars represent SD (^∗^*p* < 0.05, one-way ANOVA with *post hoc* Scheffe test).

The proximal (−1204/+183) c-Abl promoter contains a string of Sp1 sites, one of which is situated in the region where MRTF-A was detected to bind, raising the possibility that MRTF-A may interact with Sp1 to regulate c-Abl transcription. Re-ChIP assays showed that an MRTF-A-Sp1 complex was assembled on the proximal c-Abl promoter during HSC activation (Figures 4A,B). Reporter assay confirmed that co-expression of MRTF-A and Sp1 additively activated the c-Abl promoter (Figure 4C). When the Sp1 site was mutated, MRTF-A over-expression failed to activate the promoter activity (Figure 4D). The reliance of MRTF-A on Sp1 to activate the c-Abl promoter was further verified by the observation that depletion of endogenous Sp1 (Figure 4E) abolished the binding of MRTF-A to the proximal c-Abl promoter (Figure 4F). In addition, inhibition of Sp1 activity with mithramycin A also prevented MRTF-A from occupying the c-Abl promoter following PDGF stimulation (Figure 4G). Together, these data suggest that MRTF-A may directly regulate c-Abl transcription in hepatic stellate cells by forming a complex with Sp1.

### 2c-Abl Regulates MRTF-A Activity in Hepatic Stellate Cells

**FIGURE 5:**
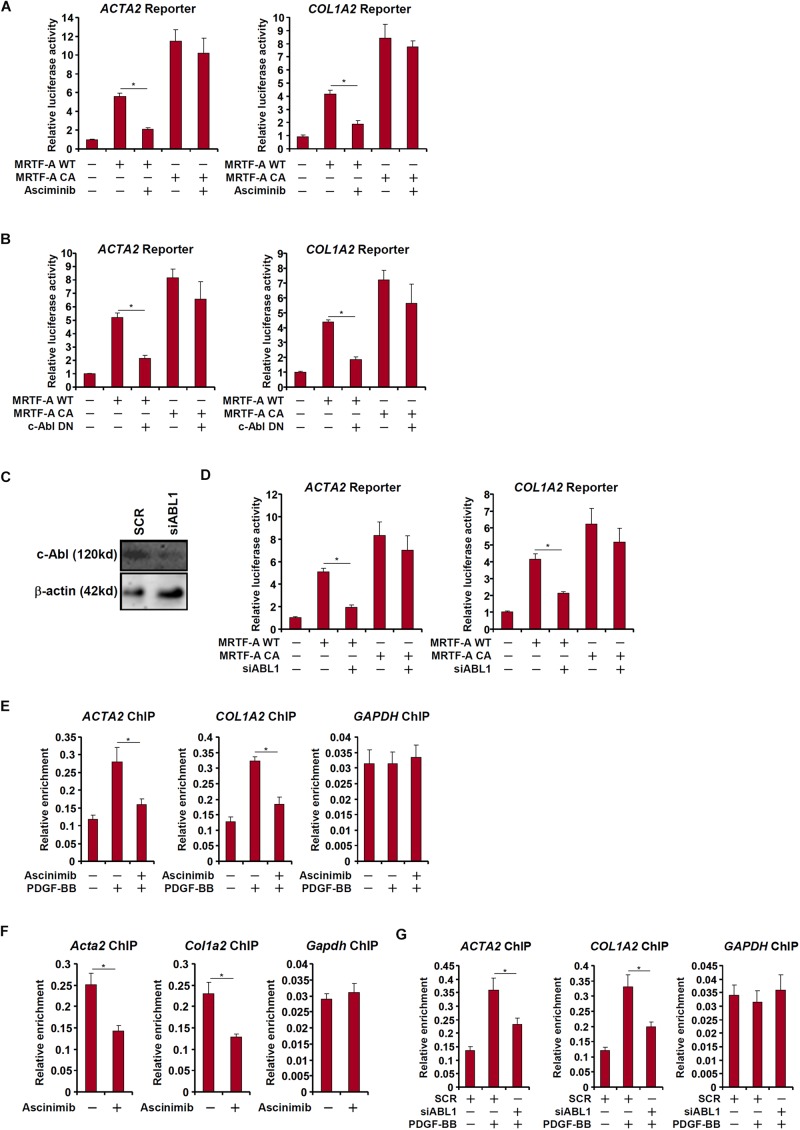
c-Abl regulates MRTF-A activity in hepatic stellate cells. **(A)** An *Acta2* reporter construct or a *Col1a1* reporter construct was transfected into LX-2 cells with or without MRTF-A followed by treatment with a c-Abl inhibitor. Luciferase activities were normalized by both protein concentration and GFP fluorescence. **(B)** LX-2 cells were treated with PDGF-BB in the presence or absence of a c-Abl inhibitor. ChIP assays were performed with anti-MRTF-A. **(C,D)** An *Acta2* reporter construct or a *Col1a1* reporter construct was transfected into LX-2 cells with MRTF-A and/or siRNA targeting c-Abl. Knockdown efficiencies were examined by Western. Luciferase activities were normalized by both protein concentration and GFP fluorescence. **(E)** LX-2 cells were treated with PDGF in the presence or absence of asciminib. ChIP assays were performed with anti-MRTF-A. **(F)** Primary murine HSCs were activated spontaneously *in vitro* for 7 days. A c-Abl inhibitor was added at day 5. ChIP assays were performed with anti-MRTF-A. **(G)** LX-2 cells were transfected with siRNA targeting c-Abl or SCR followed by treatment with PDGF-BB. ChIP assays were performed with anti-MRTF-A. All experiments were repeated three times and one representative experiment is shown. Error bars represent SD (^∗^*p* < 0.05, one-way ANOVA with *post hoc* Scheffe test).

c-Abl participates in the regulation of transcription factors by virtue of being a tyrosine kinase. Since MRTF-A activity is known to be influenced by post-translational modifications including phosphorylation (Panayiotou et al., 2016), we asked whether c-Abl could modulate MRTF-A activity either directly or indirectly. Over-expression of MRTF-A activated its target promoters (*ACTA2* and *COL1A2*) in LX-2 cells as measured by luciferase-reporter assay; treatment with a specific c-Abl inhibitor (asciminib) significantly suppressed the activities of MRTF-A target promoters (Figure 5A). Similarly, over-expression of a kinase-deficient, dominant negative (DN) c-Abl reduced the activation of the MRTF-A target promoters (Figure 5B). So did the knockdown of c-Abl (Figure 5C), which also partially relieved the activation of the MRTF-A target promoters (Figure 5D). In accordance, treatment with asciminib (Figures 5E,F) or c-Abl depletion (Figure 5G) dampened the recruitment of MRTF-A to its promoters.

### c-Abl Promotes MRTF-A Nuclear Trans-Localization in Hepatic Stellate Cells

**FIGURE 6:**
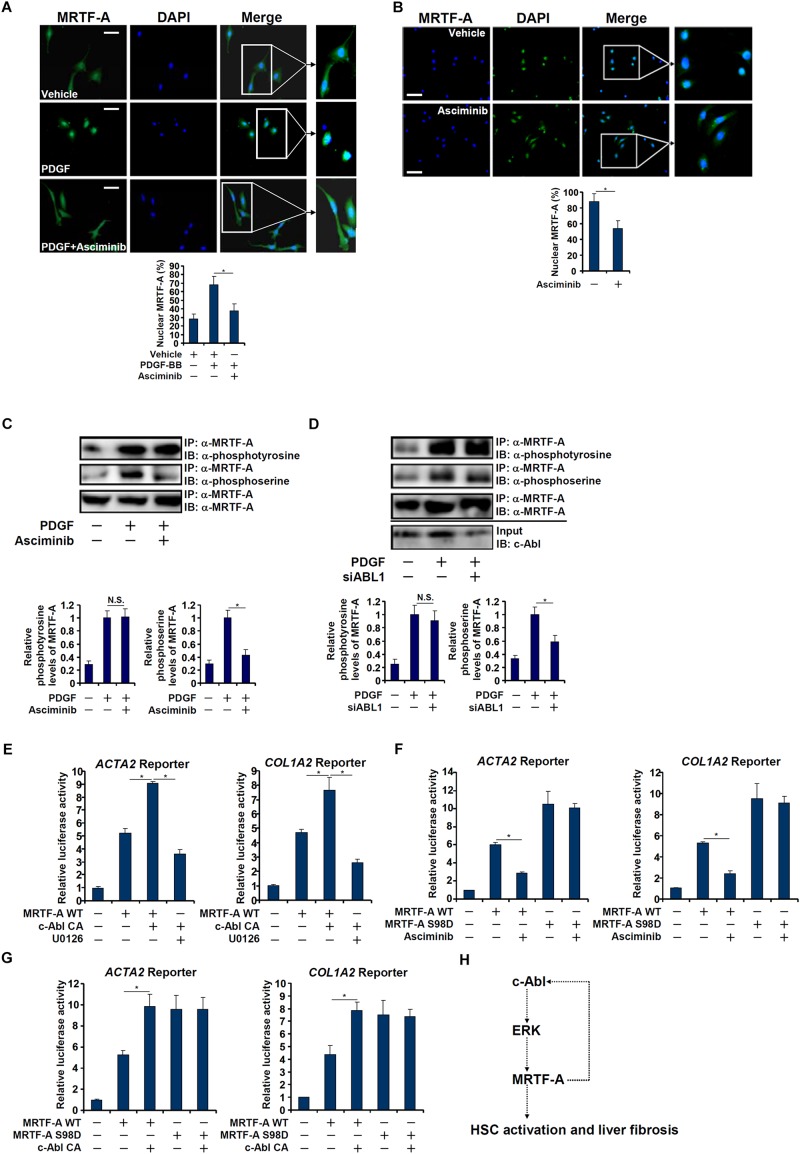
c-Abl promotes MRTF-A nuclear trans-localization in hepatic stellate cells. **(A)** LX-2 cells were treated with PDGF in the presence or absence of a c-Abl inhibitor. Nuclear MRTF-A was examined by immunofluorescence staining. Scale bar, 50 μm. **(B)** Primary murine HSCs were activated spontaneously *in vitro* for 7 days. A c-Abl inhibitor was added at day 5. Nuclear MRTF-A was examined by immunofluorescence staining. Scale bar, 50 μm. **(C)** LX-2 cells were treated with PDGF-BB and/or asciminib. Immunoprecipitation was performed with anti-MRTF-A and MRTF-A phosphorylation levels were examined by Western. **(D)** LX-2 cells were transfected with siRNA targeting c-Abl or SCR followed by treatment with PDGF-BB. Immunoprecipitation was performed with anti-MRTF-A and MRTF-A phosphorylation levels were examined by Western. **(E)** An *Acta2* reporter construct or a *Col1a1* reporter construct was transfected into LX-2 cells with WT MRTF-A and CA c-Abl followed by treatment with an ERK inhibitor. Luciferase activities were normalized by both protein concentration and GFP fluorescence. **(F)** An *Acta2* reporter construct or a *Col1a1* reporter construct was transfected into LX-2 cells with WT or S98D MRTF-A followed by treatment with a c-Abl inhibitor. Luciferase activities were normalized by both protein concentration and GFP fluorescence. **(G)** An *Acta2* reporter construct or a *Col1a1* reporter construct was transfected into LX-2 cells with WT MKL1, S98D MRTF-A and CA c-Abl. Luciferase activities were normalized by both protein concentration and GFP fluorescence. All experiments were repeated three times and one representative experiment is shown. Error bars represent SD (^∗^*p* < 0.05, one-way ANOVA with *post hoc* Scheffe test). **(H)** A schematic model.

Interestingly, a truncated MRTF-A (CA), which is constitutively kept in the nucleus (Cen et al., 2004), exhibited not only stronger trans-activity but resistance to c-Abl inhibition (Figure 5A) or deficiency (Figures 5B,D), suggesting that c-Abl might regulate MRTF-A activity via controlling its nuclear enrichment. Indeed, asciminib treatment significantly interfered with nuclear trans-localization of MRTF-A following PDGF stimulation in LX-2 cells (Figure 6A). Inhibition of c-Abl by asciminib also dampened MRTF-A nuclear importing in spontaneously activated primary mouse HSCs (Figure 6B). Of note, tyrosine phosphorylation levels of MRTF-A was not altered by c-Abl inhibition (Figure 6C) or depletion (Figure 6D) in LX-2 cells, suggesting that c-Abl might not directly use MRTF-A as a substrate. Instead, serine phosphorylation of MRTF-A was significantly down-regulated (Figures 6C,D). Previously it has been shown that serine 98 phosphorylation of MRTF-A by ERK promotes its nuclear trans-localization (Panayiotou et al., 2016). We therefore hypothesized that c-Abl might activate ERK, which in turn phosphorylates MRTF-A to facilitate nuclear transport of MRTF-A. Reporter assay showed that over-expression of a constitutive active (CA) c-Abl enhanced the activity of MRTF-A; treatment with an ERK inhibitor (U0126) blocked the effect of CA c-Abl (Figure 6E). On the other hand, suppression of MRTF-A activity by asciminib was circumvented when serine 98 was mutated to aspartate (S98D) to mimic perpetual phosphorylation (Figure 6F). Similarly, over-expression of CA c-Abl failed to further augment the S98D mutant MRTF-A (Figure 6G). Together, these data suggest that c-Abl might activate MRTF-A by promoting its nuclear trans-location in a phosphorylation-dependent manner.

## Discussion

An emerging consensus borne out of decades of rigorous investigations is that activation of hepatic stellate cells represents the key pathophysiological event in liver fibrosis regardless of its etiology. Profound changes in cellular transcriptome are associated with HSC activation (Schumacher et al., 2017). Here we detail a novel transcriptional mechanism underlying this process. We show that MRTF-A activates the transcription of c-Abl, a tyrosine kinase during HSC activation and liver fibrosis. Reciprocally, c-Abl activates MRTF-A via ERK-dependent serine phosphorylation and consequently nuclear trans-location of MRTF-A (Figure 6H). Thus, targeting this MRTF-A-c-Abl positive feedback loop may yield novel therapeutic solutions to treat liver fibrosis.

We show here that MRTF-A interacts with Sp1 to activate c-Abl transcription. Previously, it has been reported that transcription of collagen type I genes (*COL1A1*/*COL1A2*) in lung fibroblasts is regulated by an interaction between MRTF-A and Sp1 (Luchsinger et al., 2011). It has also been suggested that transcription of Slug, itself a transcription factor involved in epithelial-mesenchymal transition and fibrogenesis, is controlled by the MRTF-A/Sp1 complex (Morita et al., 2007). These results combined seem to allude to a broader theme in which MRTF-A, complexing with Sp1, orchestrates multiple transcriptional events to promote myofibroblast (HSC) trans-differentiation. Indeed, ChIP-seq experiments performed in fibroblasts demonstrate that a significant fraction of sites occupied by MRTF-A overlap with those by Sp1 although the underlying functional relevance is less clear (Esnault et al., 2014). Indeed, Sp1 blockade is synonymous with global down-regulation of ECM synthesis both *in vitro* and *in vivo* (Verrecchia et al., 2001). What remains unknown is whether and, if so, how the ability of Sp1 to regulate HSC maturation and liver fibrosis reflects its ability to engage MRTF-A as a co-activator.

MRTF-A activity is subjected to multiple layers of regulation during HSC maturation. For instance, MRTF-A protein levels, but not mRNA levels, are up-regulated as HSC trans-differentiate into myofibroblasts presumably due to miR-206 mediated suppression of MRTF-A translation (Han et al., 2017). The ability of MRTF-A to activate pro-fibrogenic transcription during HSC maturation relies on its interaction with a host of histone modifying enzymes including histone acetyltransferases and methyltransferase (Tian et al., 2015, 2016). Here we show that c-Abl may contribute to HSC trans-differentiation by promoting MRTF-A nuclear accumulation in an ERK-dependent manner. A previous profiling study by Posern and Treisman (2006) has unveiled at least 26 phosphorylation sites within MRTF-A that are dynamically regulated by serum withdrawal in NIH3T3 fibroblasts (Panayiotou et al., 2016). Although it remains to be determined whether the same MRTF-A phosphorylome dictates its activity in HSCs, suffice it to say that post-translational modification of MRTF-A serves as a key regulatory device linking specific cues to altered MRTF-A compartmentation. A few caveats exist regarding the current model. First, it is not clear whether c-Abl relies on MRTF-A phosphorylation to promote HSC activation. A wide range of c-Abl substrates, including signaling molecules, cytoskeletal proteins, and transcriptional modulators, have been identified (Wang, 2014), many of which can potentially contribute to HSC activation. For instance, phosphorylation of transcription factor STAT3 by c-Abl appears to underscore the fibrogenic process in a number of disease models (Chakraborty et al., 2017). In addition, phosphorylation of β-catenin and cyclin D by c-Abl promotes cell proliferation, a key event during HSC activation. Of interest, interplay between STAT3, β-catenin, and MRTF-A has been reported but it remains enigmatic whether c-Abl plays a role (Liao et al., 2014; Rangrez et al., 2016). Of note, c-Abl has been shown to activate RhoA GTPase (Li et al., 2015), the predominant signal required for MRTF-A nuclear translocation. It would be of great interest to decipher the c-Abl dependent protein (MRTF-A) interactome in activated HSCs. Second, the possibility that c-Abl may directly phosphorylate MRTF-A and thus regulate its activity cannot be excluded definitively. We used a pan-phosphotyrosine antibody to detect tyrosine phosphorylation of MRTF-A, which may not be sensitive enough for the subtle changes in c-Abl-dependent, site-specific tyrosine phosphorylation of MRTF-A. Further investigation is warranted to further delineate the MRTF-A-c-Abl interplay.

In summary, our data unveil a previously unrecognized route by which MRTF-A contributes to HSC activation and liver fibrosis. Considering that c-Abl inhibitors have been demonstrated to be effective in treating fibrotic diseases in pre-clinical trials (Beyer and Distler, 2013), we propose that simultaneous targeting of MRTF-A and c-Abl with dual inhibitors would prove promising in the intervention of end-stage liver diseases.

## Data Availability Statement

The raw data supporting the conclusions of this manuscript will be made available by the authors, without undue reservation, to any qualified researcher.

## Ethics Statement

The animal study was reviewed and approved by the Nanjing Medical University Ethics Committee on Humane Treatment of Experimental Animals.

## Author Contributions

YX conceived the project. YL, FL, MK, XyC, YD, XeC, and YX designed the experiments. YL, FL, MK, XyC, YD, and XeC performed the experiments and collected the data. YX wrote the manuscript. MF, DS, and YX provided funding and supervision.

## Conflict of Interest

The authors declare that the research was conducted in the absence of any commercial or financial relationships that could be construed as a potential conflict of interest.
